# Supplementary material for: Moisture availability and groundwater recharge paced by orbital forcing over the past 750,000 years in the southwestern USA
Source: Commun Earth Environ. 2024 Jul 14;5(1):376. doi: 10.1038/s43247-024-01550-0 (PMC11246950; doi:10.1038/s43247-024-01550-0)
Supplement: Supplementary file 2 — Supplementary Material [file 43247_2024_1550_MOESM2_ESM.pdf]

Supplement to:

Moisture availability and groundwater recharge paced by orbital forcing over the past 750,000 years in the southwestern USA

Simon D. Steidle[1,c], Kathleen A. Wendt[2], Yuri Dublyansky[1], R. Lawrence Edwards[3], Xianglei Li[3,4], Gracelyn McClure[3], Gina E. Moseley[1], Christoph Spötl[1]

[1]*Institute of Geology, University of Innsbruck, Innrain 52, 6020 Innsbruck, Austria*

[2]*College of Earth, Ocean, and Atmospheric Sciences, Oregon State University, 101 SW 26th Street, Corvallis, Oregon 97330*

[3]*School of Earth and Environmental Sciences, University of Minnesota, 116 Church Street SE, Minneapolis, MN 55455-0149, USA*

[4]*Institute of Vertebrate Paleontology and Paleoanthropology, Chinese Academy of Science, 142 Xizhimenwai Street, Beijing 100044, China*

[c]*correspondence: simonsteidlescience@gmail.com*

SUPPLEMENTAL MATERIAL

Types of calcite deposits and their relationship to the water table

The water table in Devils Hole undergoes diurnal fluctuations on a scale of a few centimeters due to Earth tides and weather events (Cutillo and Ge, 2006). As a result of these fluctuations, the water table oscillates within a few centimeters up and down the cave wall. This means that a small range of the cave wall alternates between being submerged in water and being exposed to air. On timescales of millennia, these small-scale oscillations are superimposed on larger-scale rises and falls of the groundwater table and are relevant for the formation of folia.

Mammillary calcite forms below the minima of diurnal water table oscillations, i.e., it is a subaqueous deposit. This speleothem type is translucent in thin section and has very few impurities and inclusions compared to folia. Its fabric is compact, comprising composite columnar crystals, each being a bundle of multiple rod-shaped crystallites (Figures S3-5). Fluid inclusions are rare (Figures S3G and S6A) and occur locally at the boundaries of adjacent bundles of crystallites (Figure S6A). Although mammillary calcite is known from other caves (also referred to as "cave clouds" – e.g., Hill and Forti, 1997; Polyak et al., 2008), the microscopic fabric described above seems to be unique to Devils Hole.

Folia occurs as mm to cm-thick and commonly porous layers in many cores. Folia is conspicuously white in reflected light and consists of mosaic to elongated calcite crystals with abundant fluid inclusions and pores ranging in size from µm to mm (Figure S5). While hiatuses locally exist within folia, the boundary to the under- and overlying mammillary calcite is often gradual (Figure S5; D'Angeli et al., 2015; Kolesar and Riggs, 2004).

Folia has been reported from several caves (e.g., D'Angeli et al., 2015; López Martínez et al., 2015). Its formation is restricted to hanging cave walls and is associated with a fluctuating water table (Kolesar and Riggs, 2004; Davis, 2012; D'Angeli et al., 2015).

Proto-folia. Thick and porous folia layers are increasingly rare in the older parts of the cores. Yet mm-thin and macroscopically white layers are sometimes present between mamillary layers. Petrographic examination of thin sections shows that the fabric of these white layers is identical to that of the mammillary calcite but contain abundant, sometimes large fluid inclusions of irregular shape (Figures S3, S4, S6B and C). Rarely, fluid inclusions contain small vapor bubbles (Figure S6B), which may be explained by either entrapment of air when temporarily emerged from water, or by entrapment of exsolved CO_2 bubbles. We refer to these layers as proto-folia, and consider them as precursors of well-developed folia. We are not aware of reports of this type of fabric in caves elsewhere.

The change from mammillary calcite to proto-folia is gradual and lacks sharp changes in the crystal fabric (Figure S3). Within a few mm, these white layers typically become more porous (Figure S3) after which a transition back to mammillary calcite occurs (Figures S1 and S2).

Cores more than 10 m above the modern water table were drilled in the near-vertical non-overhanging wall in Devils Hole #2, where the accretion of adherent particles was suppressed and therefore folia did not form. This agrees with observations in Devils Hole proper, where folia is only present where the wall is slightly overhanging (Kolesar and Riggs, 2004). Frequent alternations between submergence and exposure to air due to diurnal water table oscillations likely led to enhanced CO_2 degassing and non-equilibrium calcite precipitation conditions at wall segments located at the water/air interface, giving rise to a high abundance of aqueous inclusions and some porosity.

The abundance of proto-folia in older cores compared to proper folia in younger cores could also be related to a change of the cave microclimate associated with the gradual opening of the cave to the surface by erosion, and/or the availability of detrital particles. In summary, we interpret the proto-folia layers to reflect deposition close to the water table and thus use them as indicators of rises or falls of the paleo-water table the same way folia is used. Table S1 summarizes the main characteristics of mammillary calcite, folia and proto-folia.

Table S1: Petrographic characteristics of the three speleothem types present in Devils Hole #2 and Devils Hole proper.

	**Mammillary calcite**	**Proto-folia**	**Folia**
**Color (macroscopic)**	Translucent	White and opaque	White and opaque
**Macroscopic growth structure**	Massive and uniformly thick coating of coarsely crystalline calcite on most underwater surfaces in Devils Hole. The outer surface forms dome-shaped protuberances (Kolesar and Riggs, 2004)	mm-thin layers lacking distinct macroscopic growth structures. Only found in cores, not present at today's cave wall	Wedge-shaped in vertical section (Kolesar and Riggs, 2004)
**Fabric**	Compact fabric of composite columnar	Same fabric as mammillary	Consists of small (a few tenths of a mm to a few

	crystals, each being a bundle of multiple rod-shaped crystallites	calcite but containing much more inclusions	mm) equant to slightly elongate length-fast calcite crystals. Initial precipitates exhibit a dendritic habit, with most of the growth occurring on the upper surface of folia. With continued calcite precipitation, up to 10 mm-long columnar, length-fast calcite crystals may occur (Kolesar and Riggs, 2004)
**Boundaries**	None	Gradual changes	Sharp boundaries between different types of fabrics exist but also gradual changes
**Inclusions and pores**	Dense calcite with less than 1% porosity. Pore spaces are of two different types: (a) irregularly shaped pore spaces with fluid inclusions that are commonly oriented parallel with the crystallite boundaries. (b) Pore spaces formed by debris settling onto up-facing mammillary calcite surfaces in the plane of the crystal terminations, i.e., parallel to the growth surface (Kolesar and Riggs, 2004)	Variously abundant fluid inclusions	Abundant fluid inclusions and open pores
**Formation**	Subaqueously, below the range of diurnal water table oscillations	Mostly subaqueously but very close to the water table where enhanced degassing of CO_2 is expected to occur. Occasional exposure to air.	Within the range of water table oscillations

Data treatment, selection of outliers and subsets of data for analysis

In total, 92 samples were processed for this study. Five outliers were removed: four (N-754, N-749, J-813 and F-573) out of the five omitted samples were discarded because they are out of stratigraphic order. They are from thin layers bracketed by petrographic boundaries or rest directly on bedrock. These samples are interpreted as younger deposits whose origin is possibly related to small fractures in the calcite deposit which were later filled by calcite during a subsequent highstand. Sample G-426 was treated as an outlier and omitted because it is too old with respect to the 2-sigma uncertainty compared to G-425 and G-497 (Figure S2).

In the remaining 87 samples, the measurement of $\delta^{18}O$ and $\delta^{13}C$ failed in one case (sample N-544) and could not be repeated with similar accuracy leaving this sample without a ^{234}U-U age. Given its young age of less than the 450 ka threshold, this sample is still useful for the water table chronology but not for the comparison of both dating methods or Figure 2B.

The algorithm to derive ^{230}Th-U ages failed in 18 cases because samples are too close to secular equilibrium (entries with 0±0 ka in the supplementary data table). In nine other cases the numerical result had an age uncertainty >1000 ka. These samples are also regarded as being too close to secular equilibrium. This finally left 60 ^{230}Th-U ages, which were used (not considering the five outliers discussed above).

Given the one sample without available ^{234}U-U age (N-544), there are 59 samples where both dating methods could be successfully applied. For the second bootstrap analysis described in the main text, only samples with uncertainties <100 ka were used (i.e., 52 samples).

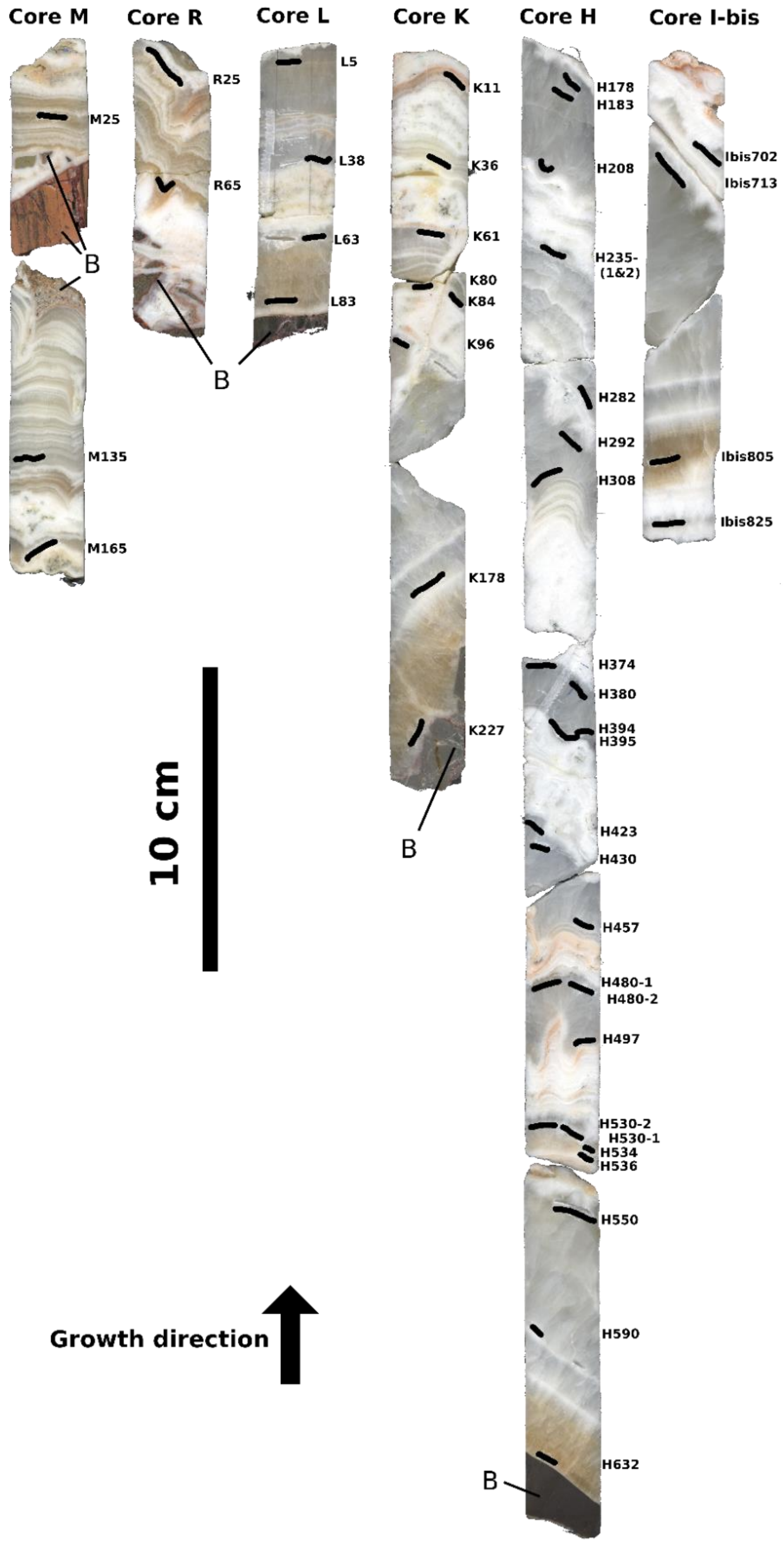

Figure S1: Cores of Devils Hole calcite drilled horizontally into the cave wall (see Figure 1). Folia and proto-folia deposits appear white while mammillary calcite appears grey and at few places brown. Bedrock and rock fragments are annotated with a "B". Black lines are drilling positions for dating of mammillary calcite (letter = name of the core; number = mm from the top of the core). Elevation above present day water table of different cores: M: 18.7m; R: 19.5m; L: 15.8m; K: 13.3m; H: 9.9m; I-bis: 8.3m. Shown are continuous segments used in this study. Top and bottom parts of cores not used in this study are not shown.

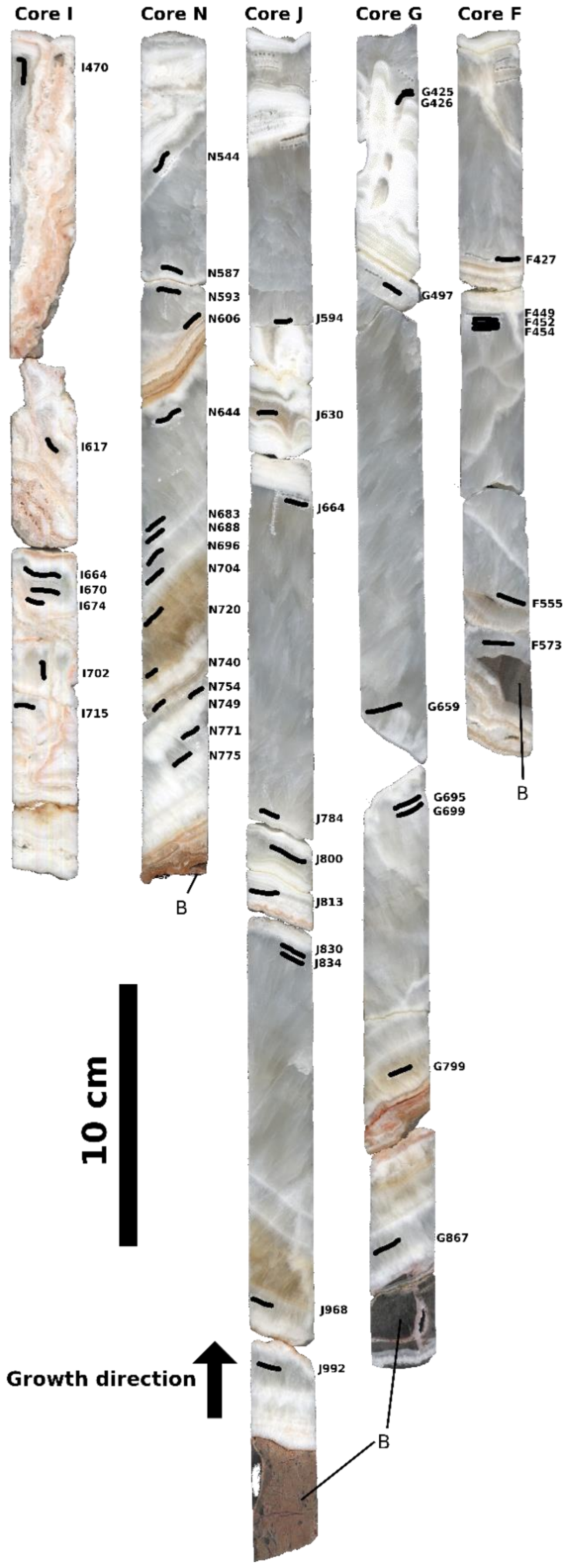

Figure S2: Cores of Devils Hole calcite (continued from Figure S1) drilled horizontally into the cave wall (see Figure 1). Folia and proto-folia deposits appear white while mammillary calcite appears grey and at few places brown. Bedrock and rock fragments are annotated with a "B". Black lines are drilling positions for dating in mammillary calcite (letter = name of the core; number = mm from the top of the core). Elevation above present day water table of different cores: I: 8.3m; N: 6.8m; J: 5.6m; G: 4.6m; F: 3.2m. Shown are continuous segments used in this study. Top and bottom parts of cores not used in this study are not shown.

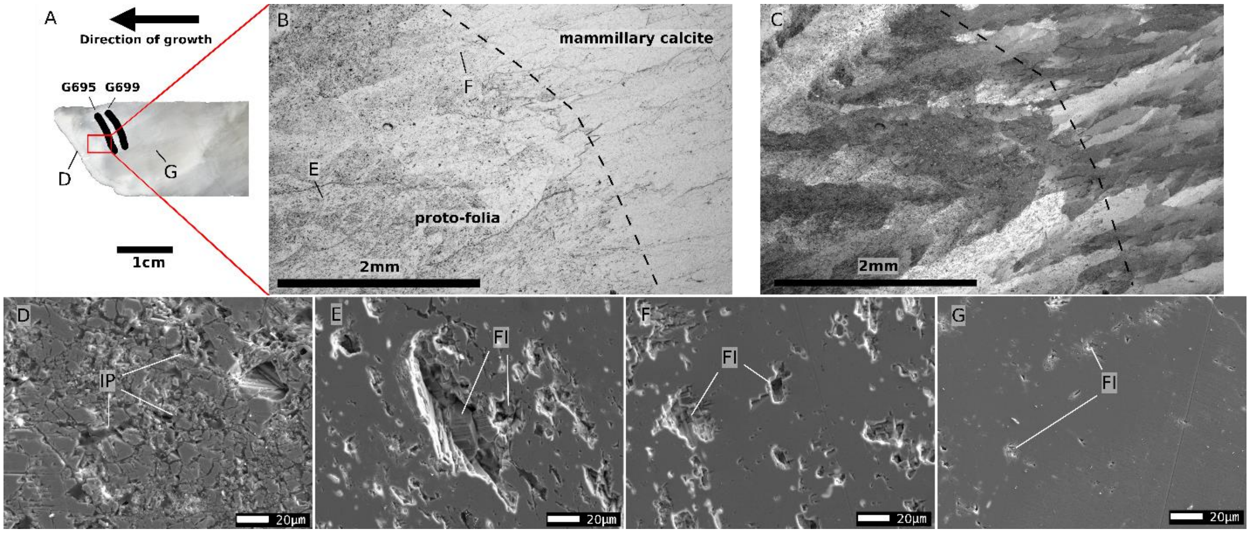

Figure S3: Transition from mammillary calcite to proto-folia. A: Overview of the middle part of drill core G (see Figure S2). Plane-polarized (B) and cross-polarized (C) transmitted-light photomicrographs showing the gradual transition from mammillary calcite to proto-folia. D-G: Scanning electron microscope images of the surface of a polished thin section showing proto-folia (D-F) and mammillary calcite (G). D – 6 mm from the mammillary calcite layer; E - 3 mm from the mammillary calcite layer; F – 1 mm from the mammillary calcite layer; G – within the mammillary calcite layer. Some pores associated with fluid inclusions (FI) and interconnected pores (IP) are marked.

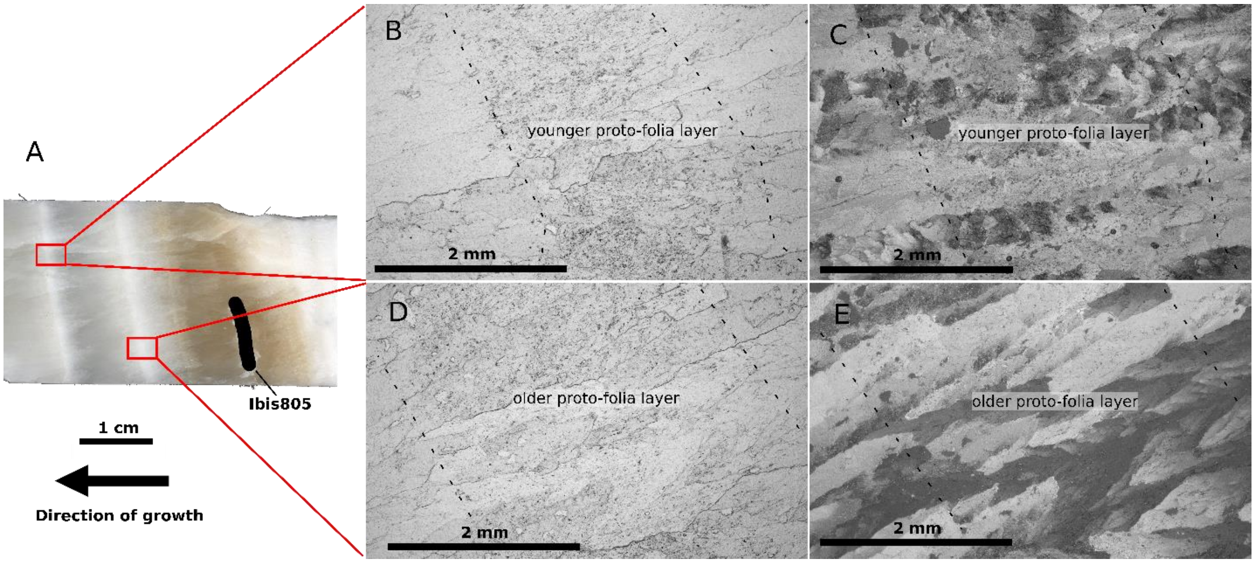

Figure S4: Two thin white proto-folia layers bracketed by mammillary calcite. A: Overview of the lower part of drill core I-bis (see Figure S1). Plane-polarized (B&D) and cross-polarized (C&E) transmitted-light photomicrographs of the younger (B&C) and older (D&E) proto-folia layer.

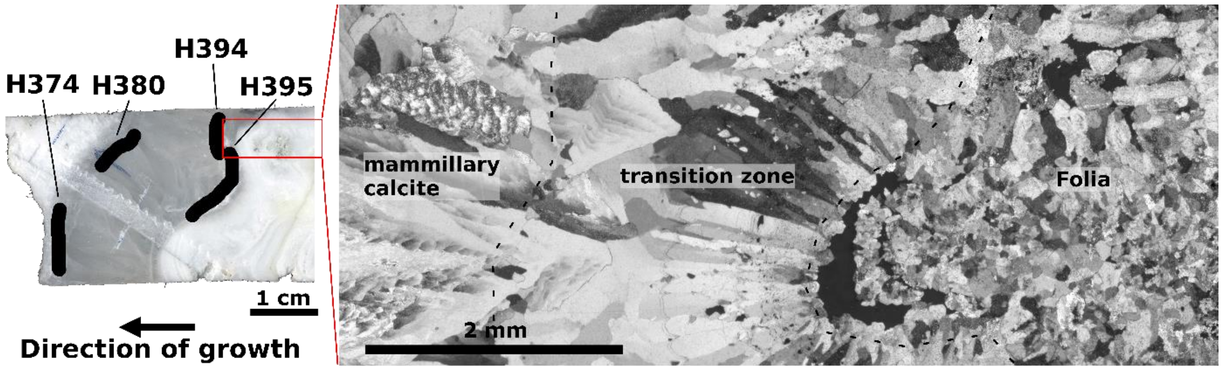

Figure S5: Transition from folia to mammillary calcite.. Left: Overview of the middle part of drill core H (see Figure S1). Right: Picture of a thin section under cross-polarized transmitted light.

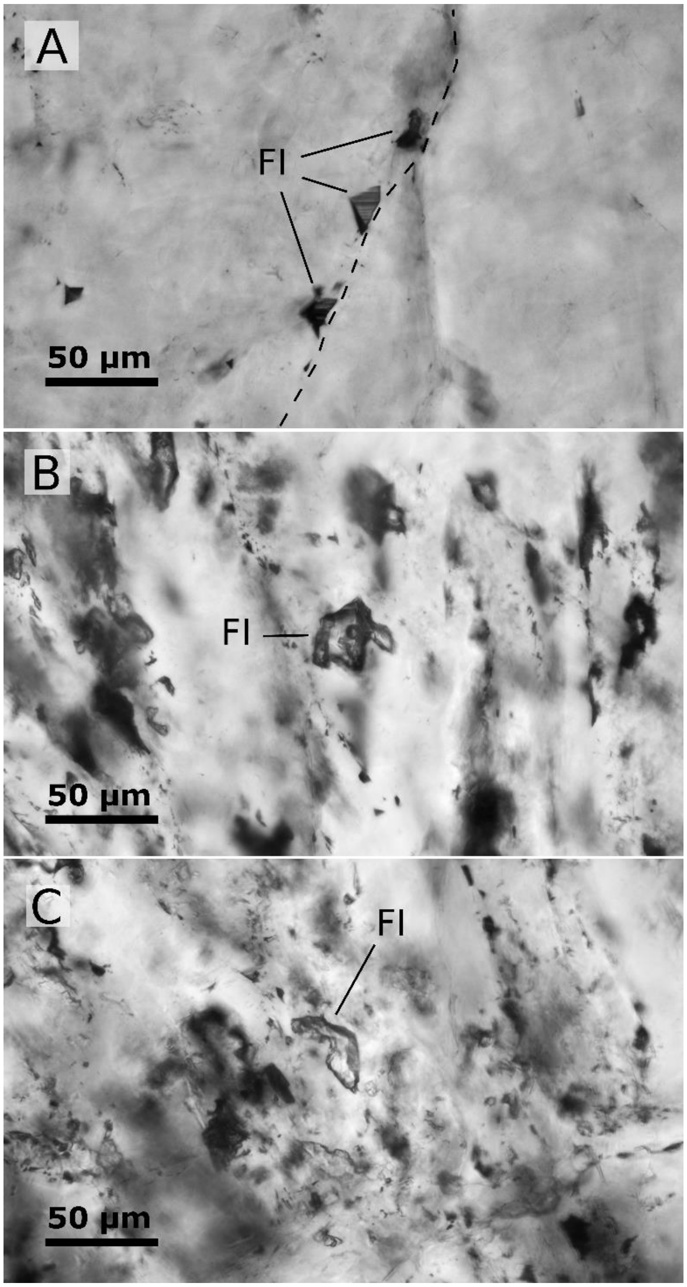

Figure S6: Fluid inclusions (FI) in mammillary calcite (A) and proto-folia (B and C) under plain-polarized light. A – three single-phase (all-liquid) inclusions alligned along the compromise boundary between adjacent crystallites (dashed line); B – relatively large two-phase (liquid-vapor) inclusion; vapor bubble was either accidentally trapped air or represents exsolved CO_2; C - relatively large single-phase (all liquid) inclusion. Many other inclusions in B and C are out of focus.

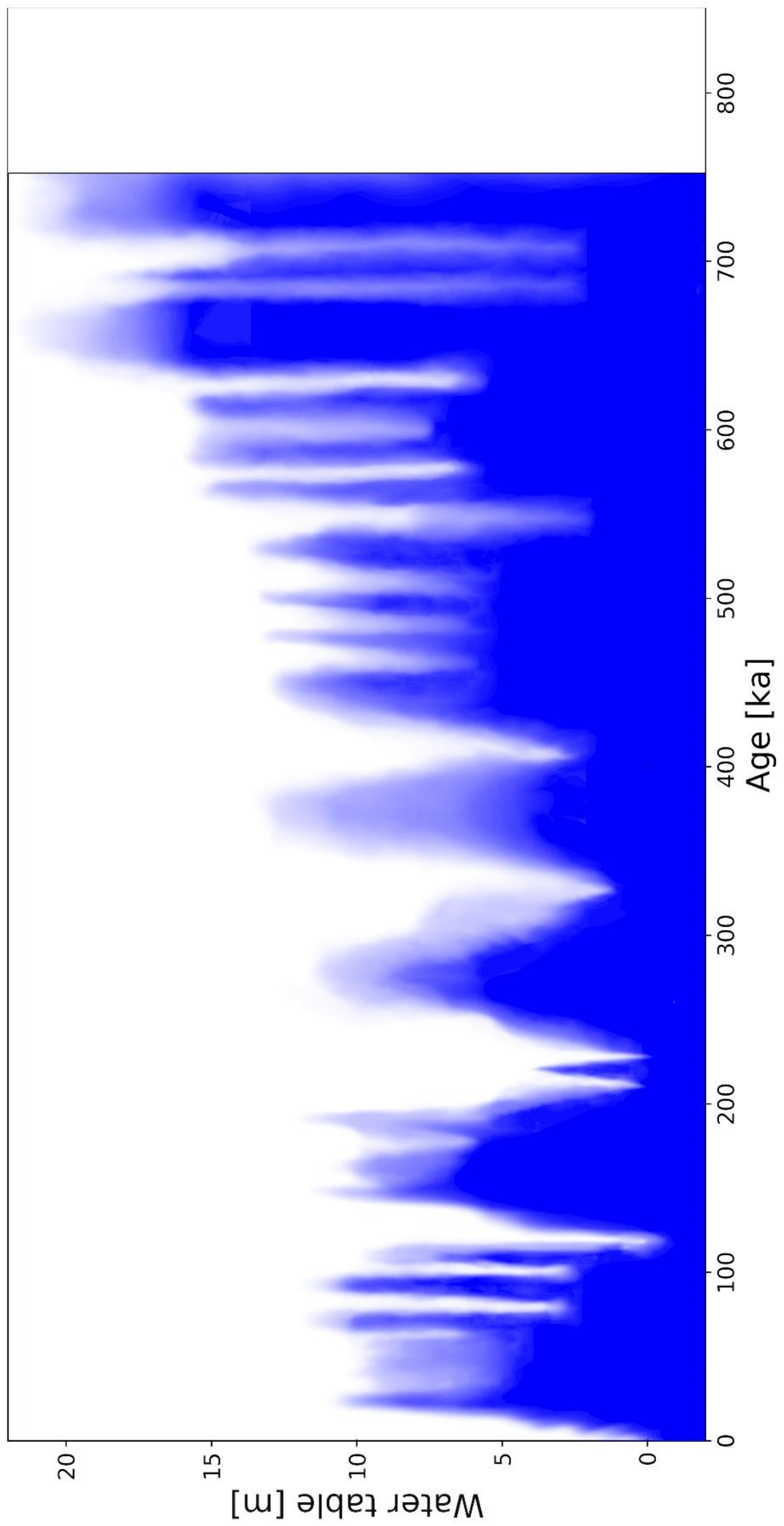

Figure S7: Visual aid of water table history (see Figure 3 and main text) used as template.

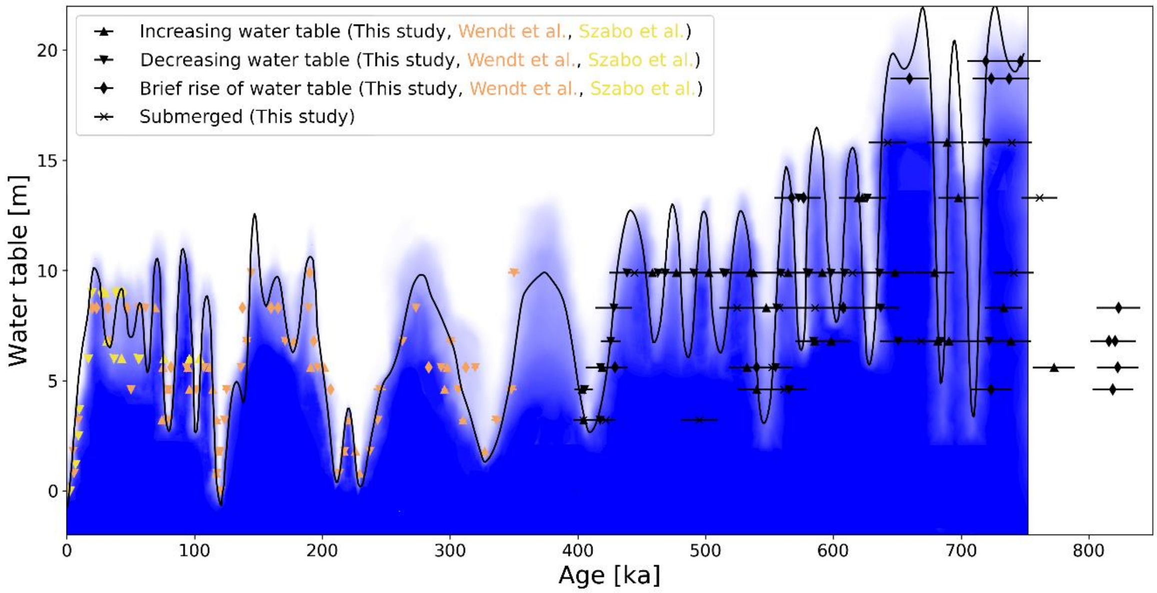

Figure S8: Spline function of the most likely water table history based on the presented water table markers. The data is attached as a supplement to the manuscript. Blue shading and water table markers are the same as in Figure 3 in the main text. Uncertainties of individual data points older than 450 ka are difficult to resolve, but they are all between ±14 ka and ±17 ka.

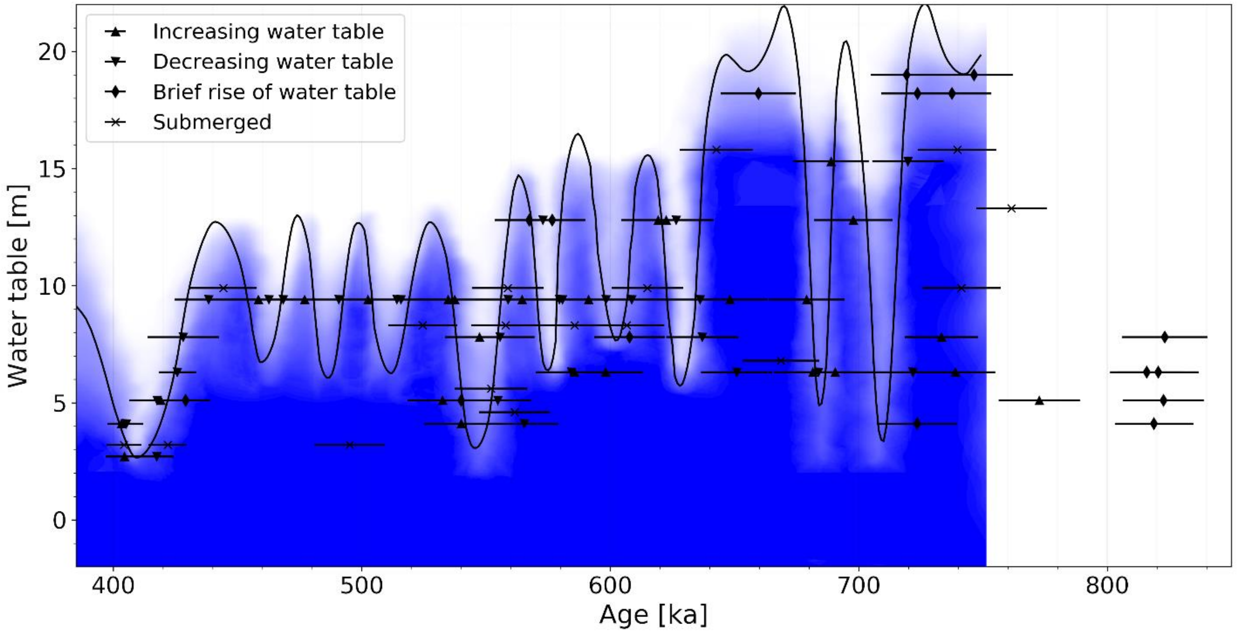

Figure S9: Water table markers and blue shading as in Figure 3 of the main text but zoomed in. Additionally, the spline function of the most likely water table history as in Figure S8. Note: Uncertainties of individual data points older than 450 ka are difficult to read, but they are all in a narrow range between ±14 ka and ±17 ka.

SUPPLEMENTARY REFERENCES

Cutillo and Ge, 2006 Analysis of strain-induced ground-water fluctuations at Devils Hole, Nevada https://doi.org/10.1111/j.1468-8123.2006.00150.x

D'Angeli et al., 2015: Genesis of folia in a non-thermal epigenic cave (Matanzas, Cuba) http://dx.doi.org/10.1016/j.geomorph.2014.09.006

Davis 2012: In defense of a fluctuating-interface, particle-accretion origin of folia http://dx.doi.org/10.5038/1827-806X.41.2.6

Hill, C., Forti, P., 1997. Cave Minerals of the World. 2nd ed., Huntsville (National Speleological Society).

Polyak, V., Hill, C., Asmerom, Y., 2008. Age and evolution of the Grand Canyon revealed by U-Pb dating of water table-type speleothems. Science, 319, 1377-1380.

Kolesar, P. T. and A. C. Riggs, "Influence of depositional environment on Devils Hole calcite morphology and petrology," in Studies of Cave Sediments, Ed.I. D. Sasowsky and J. Mylroie Springer US, 2004, pp. 227–241.

López Martínez et al, 2020: Bubble trail and folia in cenote Zapote, Mexico: petrographic evidence for abiotic precipitation driven by CO2 degassing below the water table https://doi.org/10.5038/1827-806X.49.3.2344
