# Supplementary material for: Moisture availability and groundwater recharge paced by orbital forcing over the past 750,000 years in the southwestern USA
Source: Commun Earth Environ. 2024 Jul 14;5(1):376. doi: 10.1038/s43247-024-01550-0 (PMC11246950; doi:10.1038/s43247-024-01550-0)
Supplement: Supplementary file 3 — Description of Additional Supplementary Files [file 43247_2024_1550_MOESM3_ESM.docx]

**Description of Additional Supplementary Files**

**File name:** Supplementary Data 1

**Description:** Supplementary Data (U-series data). All U-series data used in this study (both 230Th-U and 234U-U ages).

**File name:** Supplementary Data 2

**Description:** Supplementary Data (Spline function). Data of the spline function used for visual representation.
